# Microbiological Effects of Laser-Assisted Non-Surgical Treatment of Peri-Implantitis: A Systematic Review and Meta-Analysis of Randomized Controlled Trials

**DOI:** 10.3390/dj14010049

**Published:** 2026-01-12

**Authors:** Chariklia Neophytou, Elpiniki Vlachodimou, Eleftherios G. Kaklamanos, Dimitra Sakellari, Konstantinos Papadimitriou

**Affiliations:** 1 Department of Preventive Dentistry, Periodontology and Implant Biology, School of Dentistry, Aristotle University of Thessaloniki, 54124 Thessaloniki, Greece; chneophytou04@gmail.com (C.N.);; 2 School of Dentistry, European University Cyprus, Nicosia 2404, Cyprus; 3 Hamdan Bin Mohammed College of Dental Medicine, Mohammed Bin Rashid University of Medicine and Health Sciences, Dubai P.O. Box 505055, United Arab Emirates

**Keywords:** peri-implantitis, laser therapy, photodynamic therapy, diode laser, erbium laser, Er: YAG laser, Er, Cr:YSGG, microbiological outcomes, red complex bacteria, non-surgical debridement, personalized dentistry, randomized controlled trials, systematic review and meta-analysis

## Abstract

**Background**: Peri-implantitis, a condition characterized by inflammation and progressive bone loss around dental implants, presents a significant challenge in contemporary dentistry. Conventional non-surgical treatments often fail to fully eliminate bacterial biofilms, particularly on complex implant surfaces. Laser therapies have emerged as potential adjuncts due to their antimicrobial and bio-modulatory properties. However, their microbiological effectiveness and suitability for individualized patient treatment planning remain unclear. **Objective**: Τhis study aims to systematically assess and synthesize the microbiological effects of various laser-assisted non-surgical treatments for peri-implantitis compared to conventional mechanical debridement. **Methods**: This systematic review and meta-analysis followed PRISMA guidelines and was registered in PROSPERO (CRD420251035354). Randomized controlled trials (RCTs) evaluating microbiological changes following laser-assisted non-surgical treatment of peri-implantitis, with a minimum follow-up of one month, were identified through searches in multiple databases and registries up to February 2025. The ncluded studies used lasers such as diode, Er: YAG, and photodynamic therapy (PDT) either alone or as adjuncts to mechanical debridement. Outcomes of interest included bacterial counts. Risk of bias was assessed using the RoB2 tool, and certainty of evidence was evaluated via GRADE. Quantitative synthesis used random-effects meta-analysis, with standardized mean differences (SMDs) calculated. **Results**: Eight RCTs involving 266 patients and 335 implants were included in the systematic review. Quantitative synthesis of three pathogens (counts of *Fusobacterium nucleatum*, *P. gingivalis*, *T. denticola*) across three studies displayed no statistically significant differences between laser and control groups at 3 and 6 months (*p* > 0.05 for all comparisons). When examining individual study findings, PDT, particularly in patients with diabetes or acute abscess, showed short-term reductions in red complex bacteria (e.g., *Porphyromonas gingivalis* and *Treponema denticola*). In contrast, diode and Er: YAG lasers demonstrated inconsistent or transient effects. The quality of evidence was rated as very low according to GRADE. **Conclusions**: Laser-assisted therapies, especially PDT, may provide targeted microbiological benefit in selected patient groups, supporting their adjunctive use within personalized treatment planning rather than as replacements for mechanical debridement, which remains the gold standard. Further high-quality RCTs incorporating well-defined patient risk profiles, such as systemic conditions and behavioral factors, and precision treatment algorithms are needed.

## 1. Introduction

Peri-implantitis is a pathological condition affecting the tissues surrounding dental implants. It is primarily characterized by inflammation in the peri-implant mucosa and progressive loss of supporting bone [1]. It poses a significant clinical challenge in implant dentistry, with a reported prevalence ranging from 10% to 47% among implant-treated patients. Clinically affected sites often present with signs of inflammation, including bleeding on probing (BOP), suppuration, increased probing depths (PDs), and/or mucosal recession. Radiographic examinations typically show greater bone loss compared to previous radiographs [2]. If left untreated, peri-implantitis may result in implant failure, jeopardizing both patient outcomes and the longevity of implant-supported restorations. As dental implants have become increasingly popular for replacing missing teeth, managing peri-implantitis has become a major challenge in contemporary dentistry.

The primary cause of peri-implantitis is bacterial biofilm formation on implant surfaces. The microbial profile of peri-implantitis is complex and closely resembles that of periodontitis, with a predominance of anaerobic, Gram-negative bacteria. Key pathogens commonly associated with the condition include *Porphyromonas gingivalis*, *Tannerella forsythia*, and *Treponema denticola*, collectively known as the “red complex.” These bacteria possess virulence factors such as proteolytic enzymes and endotoxins and have the ability to evade host immune responses, leading to tissue inflammation and bone destruction. Other microorganisms such as *Staphylococcus aureus*, *Candida* species, *Pseudomonas aeruginosa*, and *viruses* (i.e., *Human Cytomegalovirus*, *Epstein–Barr virus*) are also frequently identified in peri-implantitis [1,3]. A comprehensive understanding of the microbiota involved is essential for guiding effective therapeutic strategies aimed at disrupting the biofilm and restoring peri-implant health.

The development and progression of peri-implantitis are further influenced by several risk indicators, including a history of chronic periodontitis, inadequate plaque control, and poor compliance with supportive periodontal care [4]. Together, these factors make peri-implantitis a multifactorial condition requiring complex preventive and therapeutic approaches.

Management of peri-implantitis typically begins with non-surgical methods aimed at reducing the microbial load and controlling inflammation. Conventional mechanical debridement (MD), using plastic or titanium curettes, ultrasonic scalers [5], or air-abrasive devices [6], serves as the first-line treatment [7]. However, these methods often fail to completely remove biofilms, particularly on the intricate geometries and rough surfaces of dental implants, resulting in suboptimal clinical outcomes. To overcome these limitations, adjunctive therapies such as antimicrobial agents [8,9], PDT, and lasers [10,11] have been investigated.

Lasers offer a range of advantages, including antimicrobial effects, precise decontamination, anti-inflammatory properties, improved access to peri-implant pockets, and potential bio-stimulation properties, all while preserving the integrity of implant surfaces and surrounding tissues [12,13,14]. These advantages suggest that laser applications could enhance the microbiological and clinical outcomes of non-surgical treatment.

Different laser systems are available, each with specific applications and limitations [15]. Carbon dioxide (CO_2_) lasers are effective for soft tissue management due to their strong absorption by water, enabling precise vaporization of inflamed tissues and excellent bleeding control [16]. However, they cannot remove biofilms or calculus, and the vaporization temperature is high, easily carbonizing the irradiated surface and limiting their use to adjunctive treatment for soft tissue management; they are not commonly employed in everyday peri-implantitis therapy.

Erbium lasers, including Er: YAG and Er, Cr:YSGG, are among the most versatile options, capable of being used as monotherapy or adjunctively. With a high affinity for water and hydroxyapatite, erbium lasers efficiently ablate biofilms, remove calculus, and detoxify implant surfaces without causing thermal damage to surrounding tissues. These properties offer an advantage for comprehensive peri-implantitis treatment, even as a stand-alone approach [17].

In contrast, diode lasers and Nd: YAG lasers are primarily used as adjuncts to MD. Diode lasers provide deep tissue penetration and bactericidal effects, while Nd: YAG lasers are particularly effective against pigmented bacteria and for modulating inflammation. However, neither diode nor Nd: YAG lasers can solely remove calculus or biofilms, necessitating their use alongside MD to achieve thorough treatment [13,18].

PDT represents another adjunctive approach, combining the light energy of a diode laser with a photosensitizer to selectively target bacteria while sparing host tissues. While minimally invasive, PDT is generally used to enhance bacterial reduction rather than as a primary treatment modality [14].

Peri-implantitis does not present uniformly across patients; microbial colonization patterns and inflammatory responses vary based on systemic conditions, immune status, lifestyle, and genetic predisposition. Therefore, non-surgical treatment outcomes may depend on individual biological profiles. Within the context of precision dentistry, identifying which patients benefit from adjunctive therapies such as lasers is essential for guiding personalized care pathways.

Despite growing interest in laser therapy for managing peri-implantitis, its microbiological efficacy remains uncertain. While some randomized controlled trials (RCTs) suggest that laser-assisted treatment leads to superior bacterial reduction and clinical improvements compared to conventional methods, others report no significant differences [12,19]. These discrepancies may result from heterogeneity in study designs, patient populations, laser types, and treatment protocols. Notably, a comprehensive synthesis focused specifically on microbiological outcomes is lacking. This systematic review and meta-analysis aims to address these gaps by critically evaluating and synthesizing existing RCT evidence on the microbiological effects of laser-assisted treatment compared to conventional non-surgical methods for peri-implantitis, with or without adjunctive antimicrobials, after a minimum healing period of one month.

## 2. Materials and Methods

### 2.1. Protocol and Registration

This review follows a protocol that was created, registered, executed, and reported in alignment with relevant methodological guidelines [20,21,22,23,24] (PROSPERO: CRD420251035354) (see Table S1. PRISMA 2020 Checklist). Since this study is a systematic review relying on existing published data, ethical approval was not required.

### 2.2. Eligibility Criteria

The eligibility criteria were based on the PICOS framework (Participants, Intervention, Comparison, Outcomes, Study design) (see Table S2. Eligibility criteria) [24].

**Participants**: Adult patients (≥18 years) diagnosed with peri-implantitis, with at least 10 participants per study.

**Interventions**: Non-surgical peri-implantitis treatment using lasers (e.g., diode, Nd: YAG, Erbium, PDT, Low-Level Laser Therapy [LLLT]), either alone or adjunctively with mechanical debridement (MD).

**Comparisons**: Conventional non-surgical treatment without lasers, including plastic or titanium curettes, ultrasonic scalers, air-abrasive devices, and/or local or systemic antimicrobials.

**Outcomes**: Microbiological changes, including quantitative shifts in peri-implant pathogens.

**Study design**: Randomized controlled trials (RCTs) with a minimum follow-up of one month.

Studies were excluded if involving animals, in vitro, ex vivo, or in silico experiments, as well as non-comparative studies (such as case reports and case series), cohort and case-control studies, non-randomized studies, systematic reviews, and meta-analyses.

### 2.3. Information Sources and Search Strategy

Searches were conducted up to 3 February 2025, across the following databases: Medline (PubMed), CENTRAL (Cochrane Library), Cochrane Database of Systematic Reviews, Scopus, Web of Knowledge (Web of Science Core Collection, KCI Korean Journal Database, Russian Science Citation Index, SciELO Citation Index, Zoological Record), and ProQuest Dissertations and Theses.

No restrictions were applied for language or publication date. Duplicates were removed using EndNote X9 (Clarivate, Philadelphia, PA, USA) and verified manually. Reference lists of included articles were also screened to identify additional studies (C.N., E.V.).

### 2.4. Study Selection and Data Collection

Two authors, C.N. and E.V., independently assessed the retrieved records for inclusion using the Rayyan platform (http://rayyan.qcri.org, accessed on 1 November 2025). The initial screening focused on titles and abstracts, eliminating studies that did not fulfill the inclusion criteria. For studies meeting these criteria or possessing unclear abstracts, the full text was reviewed to verify eligibility. From the eligible studies, C.N. and E.V. independently extracted information using predefined forms that captured: bibliographic details (title, authors, year, country, universities, open access status, funding contributions); study design and participant numbers; inclusion criteria; participant characteristics; intervention and comparator details; type and specifications of the laser used; observation period duration; bacteria types evaluated; sampling and analysis methods for bacteria; microbiological changes; and information on risk of bias assessment. Whatever disagreements occurred were resolved by a third independent reviewer (K.P.). If clarifications on published data or additional materials were needed, C.N. attempted to reach the corresponding authors via email.

### 2.5. Risk of Bias in Individual Studies

Two authors (C.N. and E.V.) independently assessed the risk of bias in individual studies using the RoB2 tool for RCT [24]. The evaluation focused on five domains: randomization process, deviations from intended interventions, missing outcome data, measurement of outcomes, and selection of reported results. These assessments were then entered into the Risk-of-bias VISualization (robvis) web application [25]. In all the processes mentioned above, any disagreements were resolved through discussions with E.G.K.; however, following the relevant suggestions, kappa statistics were not computed [24].

### 2.6. Data Synthesis and Effect Measures

Microbial outcomes were synthesized as standardized mean differences (SMDs) with 95% confidence intervals (CIs) [24,26,27]. Missing means or standard deviations were imputed using Wan et al.’s methodology [28].

A random-effects model was used to account for variability due to patient-specific and treatment factors. Heterogeneity was assessed visually (overlap of 95% CI) and statistically (τ^2^, I^2^). When possible, 95% prediction intervals were calculated to estimate likely effects in future practice. Analyses were performed in Comprehensive Meta-Analysis v3.3.070 (Biostat Inc., Englewood, NJ, USA). The significance threshold (α) was 0.05; for heterogeneity tests, α = 0.10.

Sensitivity analyses were conducted for meta-analyses with ≥3 studies, considering the following:

1. Patient medical status (diabetic vs. non-diabetic);
2. Type of mechanical debridement (manual vs. air abrasion);
3. Laser type (diode, Er: YAG, PDT, etc.

### 2.7. Reporting Bias Assessment

Potential publication bias was assessed for the meta-analysis, which was intended for ≥7 studies. Funnel plots were planned for visual evaluation [24].

### 2.8. Certainty Assessment

Evidence certainty was assessed using GRADE (Grading of Recommendations, Assessment, Development, and Evaluation) methodology [29], even when data were limited. The approach provided a structured, transparent interpretation of the evidence regarding microbiological outcomes at 6 months.

## 3. Results

### 3.1. Study Selection

**Figure 1:**
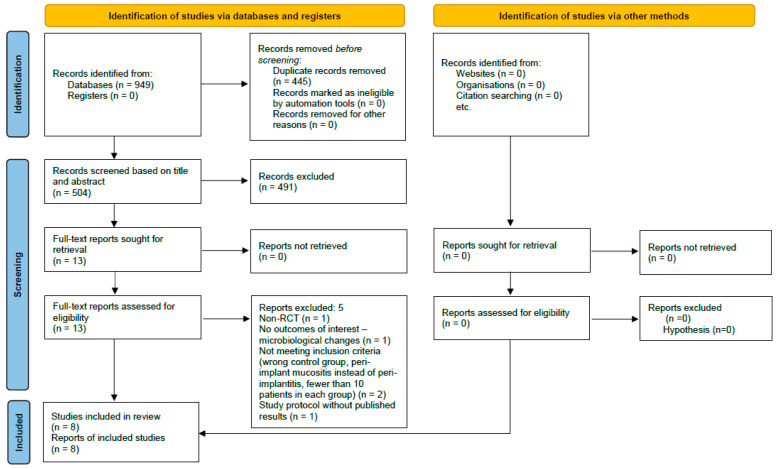
PRISMA flow diagram summarizing the study selection process.

Following a comprehensive search of electronic databases, 949 records were identified. After removing 445 duplicates, an additional 491 records were excluded based on title and/or abstract screening. Thirteen full-text articles were assessed for eligibility, five were excluded for the following reasons: one was not an RCT; one did not report microbiological outcomes; one focused on peri-implant mucositis rather than peri-implantitis; one had a sample size < 10 patients; and one was a study protocol without published results; despite attempts to contact the authors (see Table S3. Detailed strategy for database search). Ultimately, eight studies were included in the systematic review [30,31,32,33,34,35,36,37] (Figure 1).

### 3.2. Study Characteristics

The characteristics of the included studies, published between 2011 and 2022, are summarized in Table 1 and Supplementary Table S4. Additional information about the included studies. All were RCT; seven employed a parallel-arm design, while one study [31] utilized a split-mouth approach. The trials were conducted across diverse geographic locations, including Sweden, Turkey, Saudi Arabia, Taiwan, the USA, Germany, and Switzerland. In total, the studies assessed 266 patients and 335 implants, with individual sample sizes ranging from 10 to 48 patients.

The test groups received a variety of laser-based interventions, such as Er: YAG, diode, and PDT (sometimes combined with LLLT or local antimicrobials).

Lasers were used by Persson et al. [30] and Chen et al. [33], employing settings of 100 mJ/pulse and 10 Hz in pulsed mode, with decontamination-optimized tip types and exposure durations. Diode lasers (810 nm) were applied by Arısan et al. [31], Labban et al. [34], and Roccuzzo et al. [32], with notable variation in parameters: Arısan used 1 W in pulsed mode for 1 min with a 400 μm fiber tip; Labban applied 200 mW for 30 s at the papilla and 10 s in the pocket; Roccuzzo delivered 2.5 W in pulsed mode, repeated three times for 30 s [31,32,34]. PDT was employed in studies, using photosensitizers such as phenothiazine chloride, indocyanine green (ICG), or methylene blue, activated by diode lasers (660–810 nm) with short exposure times (10–30 s per site) [34,36,37]. Tribble et al. utilized a combined approach of PDT with LLLT and a photosensitizer, referred to as antimicrobial PDT (aPDT) [35].

The control groups received conventional MD with ultrasonic scalers, plastic or titanium curettes, sometimes combined with adjunctive treatments. For example, Persson et al. [30] and Bassetti et al. [37] used air-abrasive systems (e.g., Perio-Flow, glycine powder). In Almohareb et al. [36], systemic antibiotics (amoxicillin 500 mg and metronidazole 400 mg, three times daily for seven days) were administered in the control group. In Bassetti et al. [37], minocycline microspheres were locally applied and reinserted when bleeding on probing (BoP) persisted.

Microbiological assessment methods varied, and the number of species evaluated often focused on red complex bacteria. In most trials, subgingival plaque was collected from the deepest peri-implant pocket using sterile paper points. Persson et al. [30] applied checkerboard DNA–DNA hybridization to analyze 74 bacterial species, while other studies used polymerase chain reaction (PCR), quantitative real-time PCR (qPCR), anaerobic cultures, or 16S rRNA gene sequencing (e.g., Chang et al. [33] to quantify bacterial load and composition. The number of bacterial species evaluated ranged from two (e.g., *P. gingivalis* and *T. denticola* in Labban et al. [34]) to over seventy in Persson et al. [30]. Several studies focused on red complex bacteria (*P. gingivalis*, *T. denticola*, *T. forsythia*), along with other relevant pathogens, such as *Fusobacterium nucleatum*, *Aggregatibacter actinomycetemcomitans*, *Campylobacter rectus*, *Eubacterium nodatum*, and *Prevotella intermedia*.

Follow-up periods ranged from 1 to 12 months, with short-term follow-up (1–3 months) being more common. However, three studies extended follow-up to 12 months [33,36,37].

In addition to microbiological outcomes, some studies assessed clinical, radiographic, and biochemical markers of inflammation. Commonly reported clinical parameters included probing depth (PD), clinical attachment level (CAL), bleeding on probing (BoP), plaque index (PI), width of keratinized tissue (KT), and marginal bone loss (MBL) or percentage bone loss. Inflammatory biomarkers such as interleukins (IL-1β, IL-6, IL-10) and matrix metalloproteinases (MMP-1, MMP-8) were also analyzed in some trials [32,34,37].

All included studies reported comparable baseline clinical characteristics between groups, and most justified their sample sizes. Funding sources varied: some studies were supported by academic institutions (e.g., Istanbul University, Southern Taiwan Science Park), while others received commercial sponsorship (e.g., EMS, Bredent Medical GmbH). Several studies were open access, facilitating transparency and reproducibility.

### 3.3. Risk of Bias Within Studies

**Figure 2:**
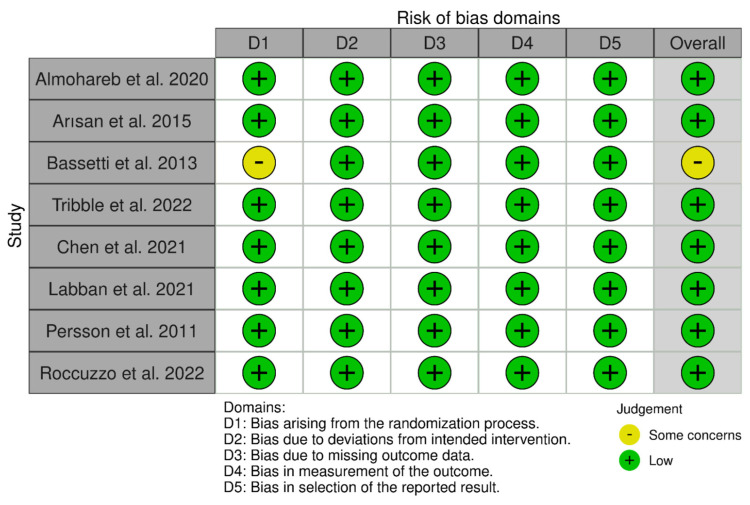
Risk of bias assessment (using the Cochrane RoB 2 tool) of the included studies [30,31,32,33,34,35,36,37].

Figure 2 summarizes the risk of bias in the included studies. All studies were assessed with RpB2 to have a low overall risk of bias, except for the study by Bassetti et al. [37], which raised some concerns mainly about the randomization process. While this study stated that it was a randomized clinical trial, it failed to provide details on the method of randomization (Question 1.1) or on whether allocation concealment was achieved (Question 1.2). We reached out to the corresponding authors for clarification, but no further information was received. Despite the absence of methodological specifics, there were no baseline differences noted between the intervention groups (Question 1.3), indicating that potential bias from the randomization process may have been minimal. The other seven studies were classified as having a low risk of bias in this area, having clearly outlined their randomization and allocation concealment methods. These methods included computer-generated randomization software [30,33], a coin toss [31,36], block randomization [34], sealed envelopes [34,36], and random number tables [32,33].

All eight studies were assessed to have a low risk of bias concerning deviations from intended interventions. Although blinding participants and personnel was impractical due to the inherently diverse nature of the interventions (e.g., MD, laser therapy, air-abrasive devices, or local/systemic antimicrobials), there is no indication that the lack of blinding resulted in deviations from intended interventions specifically due to the trial context (Question 2.3). Notably, two studies [32,33] used partial blinding for participants by employing inactive or non-activated laser light as a placebo. In Tribble’s study, the control group received a sham light treatment meant to simulate adjunctive antimicrobial photodynamic therapy [35]. Likewise, in Roccuzzo’s study, a non-activated diode laser was utilized at control implant sites [32]. However, in both instances, while participants were blinded, the personnel administering the interventions were not. Throughout all studies, there were no reported deviations from the intended interventions that would likely have impacted study outcomes (Question 2.4), nor any evidence suggesting that potential deviations were unevenly distributed among intervention groups (Question 2.5). Importantly, all studies employed ITT analyses (Question 2.6), and there were no concerns regarding exclusion or misallocation of participants (Question 2.7), further supporting a low risk of analytical bias in this area.

Regarding the absence of outcome data, all studies were assessed as having a low risk of bias, despite some minor participant attrition. For instance, Labban et al. noted 1 out of 49 participants lost to follow-up at the 3-month assessment [34]; Chen et al. reported a withdrawal of one participant from the test group after 4 weeks [33]; Chang indicated that two participants missed the 12-week follow-up without giving reasons; and Bassetti mentioned two participants who did not attend the 9- and 12-month follow-ups [37]. In the study by Roccuzzo et al., 5 out of 30 participants (16.67%) were lost to follow-up (3 from the test group and 2 from the control group) due to unwillingness to continue participation [32]. Although this percentage is relatively high, the reasons for attrition across all studies were not related to the interventions themselves, and the missing data do not seem to correlate with the actual outcome values. Therefore, the overall risk of bias due to missing data is considered low in all studies.

All studies were evaluated as having a low risk of bias concerning the measurement of outcomes. The methods used were suitable for the intended results (Question 4.1), and there was no evidence suggesting that outcome measurement varied among the intervention groups (Question 4.2). In every study, the assessors of outcomes were blinded to the participants’ intervention status (Question 4.3), which minimized the risk of measurement bias. This blinding decreases the chances that the results of outcome assessments were influenced by knowledge of the intervention received (Questions 4.4 and 4.5).

Furthermore, there was no indication of selective reporting bias. All studies seemed to follow pre-established analysis plans that were set before the outcome data were unblinded (Question 5.1). The results reported aligned with the outcomes and analyses specified in the protocols, and there was no sign of selective outcome reporting (Questions 5.2 and 5.3). Consequently, this domain was evaluated as having a low risk of bias across all included studies.

### 3.4. Results of Individual Studies

The following summarizes the microbiological outcomes observed for each type of laser intervention across the included studies:

**Er: YAG lasers**: Inconsistent microbiological effects; no significant long-term reductions in anaerobic counts were observed [30,33].

**Diode lasers**: Early transient reductions in some bacteria, but generally unsustained by 6 months [31,32].

**PDT**: More consistent reductions in red complex bacteria, particularly in diabetic patients, but not always statistically significant [34,35,36]. Adjunctive antimicrobials enhanced sustained effects [36,37].

To be more specific, Er: YAG lasers were evaluated as standalone interventions in two studies due to their unique ability to remove calculus and exert antimicrobial effects. However, their microbiological efficacy proved inconsistent. Persson et al. reported no significant bacterial reductions at six months among the 74 species analyzed [30]; notably, greater short-term (1 month) reductions were observed in the control group, which received air-abrasive therapy. Similarly, Chen et al. found no significant long-term improvements at six months in anaerobic microbial counts following repeated Er: YAG laser applications [33].

The microbiological outcomes of diode laser therapy were also largely inconclusive. Arısan et al. (2015), who evaluated 20 bacterial species, observed no significant reductions one month post-treatment with an 810 nm diode laser compared to MD alone [31]. Roccuzzo et al. reported early reductions in bacterial levels at three months in both test and control groups; however, by six months, only *F. nucleatum* remained suppressed, and exclusively in the MD group, suggesting that any laser-related microbial effects were transient and unsustained [32].

In contrast, PDT, particularly as an adjunctive treatment, demonstrated more consistent and favorable microbiological outcomes. Labban et al. reported significant reductions in *P. gingivalis* and *T. denticola* at three and six months in diabetic patients treated with repeated PDT sessions using ICG as the photosensitizer [34]. Similarly, Tribble et al. observed reductions in red complex bacteria in both the PDT and MD groups, though these changes did not reach statistical significance [35].

Additional support for PDT was observed in studies incorporating adjunctive antimicrobials. Bassetti et al. (2013) found that both PDT and LDD with minocycline significantly reduced red complex bacteria, with LDD producing more sustained effects over 12 months [37]. Almohareb et al. (2020) demonstrated that PDT achieved microbial reductions comparable to those seen with systemic antibiotics, particularly against *P. gingivalis*, *T. denticola*, and *T. forsythia* [36]. These findings highlight PDT’s potential as a non-antibiotic alternative in the management of peri-implantitis.

### 3.5. Results of Synthesis

Due to heterogeneity, the meta-analysis included only three studies [30,32,34] and three pathogens (*F. nucleatum*, *P. gingivalis*, *T. denticola*). This limited dataset reduces generalizability.

***F. nucleatum***: Pooled SMD −0.044 (95% CI: −0.525 to 0.436; *p* = 0.856) at 3 months, 0.132 (95% CI: −0.348 to 0.613; *p* = 0.590) at 6 months.

***P. gingivalis***: Pooled SMD −1.034 (95% CI: −2.863 to 0.796; *p* = 0.268) at 3 months, −0.476 (95% CI: −1.444 to 0.492; *p* = 0.335) at 6 months.

***T. denticola***: Pooled SMD −3.196 (95% CI: −9.237 to 2.846; *p* = 0.300) at 3 months, −1.559 (95% CI: −4.994 to 1.876; *p* = 0.347) at 6 months.

**Figure 3:**
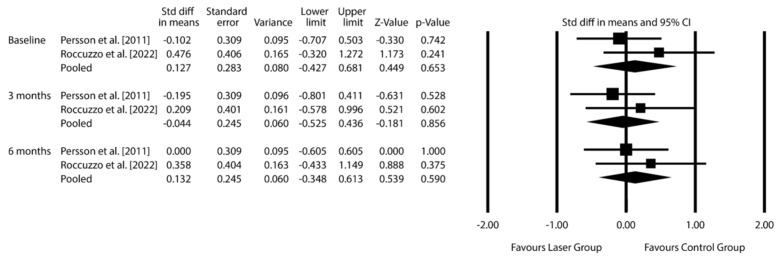
Effect of laser therapy on *Fusobacterium nucleatum* count changes at 3 and 6 months, expressed as standardized mean differences (SMD), compared to conventional non-surgical treatment for peri-implantitis. [CI = confidence interval] [30,32].

**Figure 4:**
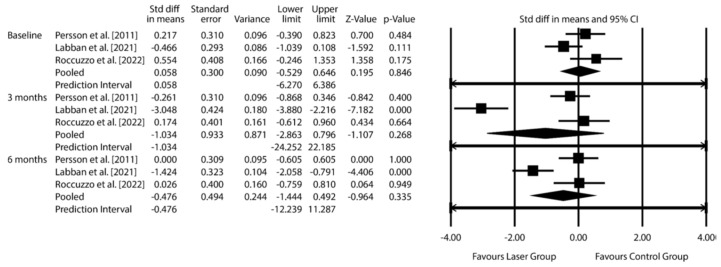
Effect of laser therapy on *Porphyromonas gingivalis* count changes at 3 and 6 months, expressed as standardized mean differences (SMD), compared to conventional non-surgical treatment for peri-implantitis. [CI = confidence interval] [30,32,34].

**Figure 5:**
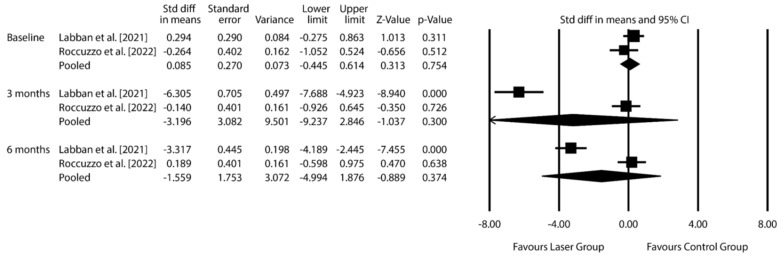
Effect of laser therapy on *Treponema denticola* count changes at 3 and 6 months, expressed as standardized mean differences (SMD), compared to conventional non-surgical treatment for peri-implantitis. [CI = confidence interval] [32,34].

The exploratory data synthesis was conducted using microbiological data from three RCTs involving control groups subjected to MD alone: [30,32,34]. The analysis focused on three periodontal pathogens strongly associated with peri-implantitis: *F. nucleatum*, *P. gingivalis*, and *T. denticola*. Results are presented in Figure 3, Figure 4 and Figure 5 and Figures S1–S3 (Additional meta-analytic data on *Fusobacterium nucleatum*; *Porphyromonas gingivalis*; *Treponema denticola*).

For *F. nucleatum*, data from Persson et al. and Roccuzzo et al. at 3 and 6 months revealed no statistically significant differences between the laser-assisted treatment groups (Er: YAG and diode) and control groups. The pooled standardized mean difference (SMD) was −0.044 (95% CI: from −0.525 to 0.436; *p* = 0.856) at 3 months and 0.132 (95% CI: from −0.348 to 0.613; *p* = 0.590) at 6 months, indicating no benefit from adjunctive laser therapy.

Analysis of *P. gingivalis* included three studies [30,32,34]. The pooled SMD was −1.034 (95% CI: from −2.863 to 0.796; *p* = 0.268) at 3 months and −0.476 (95% CI: from −1.444 to 0.492; *p* = 0.335) at 6 months—again, not statistically significant.

For *T. denticola*, data from Labban et al. and Roccuzzo et al. yielded an SMD of −3.196 (95% CI: from −9.237 to 2.846; *p* = 0.300) at 3 months and −1.559 (95% CI: from −4.994 to 1.876; *p* = 0.347) at 6 months, further supporting the lack of significant microbial improvement attributable to laser therapy [32,34].

Sensitivity analyses based on debridement modality (air-abrasion versus conventional MD) did not alter the overall findings. However, subgroup observations suggested potentially greater microbial reductions in diabetic patients and in those receiving PDT. These trends may be influenced by patient-specific systemic conditions, particularly diabetes (see Table S5. Sensitivity analyses).

A meta-analysis was not possible for studies involving control groups with MD and microbiological agents (such as systemic antibiotics or local antimicrobials) due to significant methodological differences.

### 3.6. Reporting Bias Assessment

Due to the small number of studies, a formal assessment of publication or reporting bias was not feasible.

### 3.7. Certainty Assessment

The evaluation of evidence certainty and quality regarding the findings from the exploratory data synthesis on microbial count variations following laser-assisted non-surgical peri-implantitis treatment at 6 months, using the GRADE methodology, revealed a very low certainty of evidence for all outcomes (Table 2). While the evidence is sourced from randomized controlled trials and the risk of bias and publication bias were not deemed significant, the overall quality was lowered due to serious concerns over inconsistency, indirectness, and imprecision. These issues reflect variability in results, major differences in study populations, and wide confidence intervals, which compromise the applicability and reliability of the findings.

## 4. Discussion

This systematic review and meta-analysis aimed to evaluate the microbiological effects of laser-assisted, non-surgical peri-implantitis treatment compared to MD. Although various laser modalities, including Er: YAG, diode lasers, and PDT, were studied, the pooled data indicate that there is no statistically significant microbiological advantage of laser-assisted therapies over MD.

The findings of this review emphasize that the effectiveness of laser-assisted peri-implantitis therapies is likely patient-dependent rather than universally applicable. Variation in microbiological response was most apparent in individuals with systemic conditions such as diabetes and in cases presenting with acute inflammation or abscess formation. This underscores that microbiological outcomes under treatment are influenced by patient-specific host and disease phenotypes, reinforcing the importance of personalized, individualized clinical strategies.

Among these modalities, PDT emerged as the most promising adjunctive therapy, particularly in systemically compromised patients or those presenting with acute abscesses, warranting further investigation. Individual studies, such as those by Labban et al. and Almohareb et al., reported significant short-term reductions in key pathogens, including *P. gingivalis*, *T. denticola*, and *T. forsythia* [34,36]. However, these reductions were inconsistently sustained beyond six months, as reflected in meta- analysis, which showed no statistically significant differences between laser-assisted and control groups in the reduction of *F. nucleatum*, *P. gingivalis*, or *T. denticola*.

In contrast, Er: YAG and diode lasers demonstrated minimal or transient microbiological benefit, possibly due to suboptimal laser–tissue interactions, variability in delivery protocols, and the inherent resistance of complex biofilms to laser-mediated disruption.

Clinical, radiographic, and inflammatory outcomes from the same studies offer important context. For example, Persson et al. and Chen et al. found that MD groups experienced superior PD reduction and CAL gain despite the absence of microbiological superiority [30,33]. Arısan et al. reported greater MBL in the diode laser group, while Roccuzzo et al. noted more sustained bacterial suppression in the MD-only group. Labban et al., although showing bacterial reduction with PDT, observed a rebound in inflammatory markers (e.g., IL-1β), highlighting the potential for disease recurrence [31,32,34]. These findings suggest that microbiological outcomes alone may not fully reflect overall treatment effectiveness.

The overall quality of microbiological evidence was rated as very low according to GRADE, reflecting substantial uncertainty about the effect of laser-assisted therapies. This limitation restrict the generalizability and reinforces the need for cautious interpretation of the result. Future high-quality RCTs with standardized protocols and extended follow- up are necessary to clarify the role of lasers in peri-implant disease management.

### 4.1. Interpretation of Findings

MD remains the cornerstone of non-surgical peri-implantitis management. Although laser-based therapies have been explored as potential adjuncts, their additive microbiological effect appears modest, inconsistent, and largely patient-specific.

Among the studied modalities, PDT demonstrated the greatest potential—particularly when used adjunctively in medically compromised patients or those with acute infections. Several individual studies reported reductions in red complex pathogens such as *P. gingivalis* and *T. denticola* following PDT [37], with more pronounced effects observed in medically compromised patients, such as those with diabetes [34] or acute infections [36]. Nonetheless, even in these favorable cases, the meta-analysis showed that pooled microbial outcomes did not reach statistical significance, reflecting the lack of consistent long-term microbiological benefit.

By contrast, Er: YAG and diode lasers—either as monotherapies or adjuncts—did not demonstrate consistent superiority over MD, with negligible or non-significant microbial changes in most studies (e.g., [30,31,33]). Several factors may explain this limited efficacy, including wavelength-specific tissue interactions, insufficient penetration depth for effective bacterial elimination, and variability in treatment protocols or operator technique.

The meta-analytic results further reinforced these findings, as pooled estimates for *F. nucleatum*, *P. gingivalis*, or *T. denticola* revealed no statistically significant differences between test and control groups. Although some individual trials observed short-term microbial reductions, these effects were not sustained at 3–6 months, emphasizing the importance of ongoing maintenance and possible retreatment strategies.

Regarding methodological quality, most included studies demonstrated a low risk of bias. Apart from one trial with unclear randomization procedures, the majority adhered to robust design elements, including adequate allocation concealment, low attrition, protocol fidelity, and blinded outcome assessment. Consistent outcome reporting further supports the internal validity of this review.

### 4.2. Clinical Implications

From a clinical standpoint, laser-assisted therapy, particularly PDT, may offer short-term microbiological improvements in specific patient populations. These include early-stage mucositis, individuals with systemic conditions such as diabetes mellitus, cases involving acute symptoms (e.g., abscesses), or patients for whom antibiotic use is contraindicated.

The potential of PDT to reduce or replace systemic antibiotic use is particularly relevant given global concerns about antimicrobial resistance. However, current evidence does not support the routine use of laser-based therapies as a superior alternative to MD. Instead, lasers should be considered adjunctive tools within a personalized treatment strategy where systemic health, patient preferences, and antimicrobial stewardship are central considerations.

The transient nature of observed microbiological improvements underscores the importance of integrating laser therapy into a broader supportive care framework, including individualized recall intervals, meticulous biofilm control, and, where appropriate, adjunctive host-modulation therapies to maintain peri-implant health.

### 4.3. Comparison with the Existing Literature

The findings of the present review align with prior systematic reviews examining the adjunctive use of laser therapy in the management of both periodontal and peri-implant diseases, highlighting modest and often inconclusive microbiological and clinical effects.

Previous reviews reported limited microbiological benefits and transient clinical improvements with diode and other lasers. A 2020 systematic review [38] investigating diode laser therapy as an adjunct in the non-surgical treatment of peri-implant mucositis suggested a potential to reduce PI and early clinical indicators of inflammation, such as BoP. However, it emphasized that microbiological improvements were limited and clinical benefits were typically transient.

Earlier evidence from the periodontal literature, including a 2008 systematic review [39], similarly reported that laser therapy showed promising but inconsistent results when used adjunctively with non-surgical periodontal treatment. This review found no consistent evidence supporting the efficacy of lasers in adults with chronic periodontitis and concluded that further RCTs were necessary.

Another systematic review [40] focused on laser therapy in the treatment of peri-implant mucositis and peri-implantitis, reporting some short-term clinical improvements—especially in BoP—but minimal impact on PD, CAL, REC, or PI, relative to mechanical approaches.

More recently, a 2022 systematic review focusing on radiographic outcomes [41] suggested that laser therapy may promote bone gain and reduce PD and BoP, with effects comparable to MD. However, this review noted a lack of statistically significant findings and did not evaluate microbiological outcomes.

In contrast, the present review addresses a critical gap by focusing specifically on microbiological outcomes, which have been underreported in the previous literature. By incorporating recent RCTs that employed advanced microbial analysis techniques—such as qPCR and 16S rRNA gene sequencing—this study offers a more detailed synthesis of bacterial changes associated with various laser modalities. To the best of our knowledge, it is the first systematic review to quantitatively analyze microbiological data from RCTs in the context of peri-implantitis.

### 4.4. Strengths and Limitations

This review presents several notable strengths. It follows a rigorous methodological framework based on PICOS criteria and includes only RCTs, which represent the highest level of evidence for therapeutic efficacy. It is also the first systematic effort to quantitatively assess microbiological changes associated with various laser modalities in the non-surgical management of peri-implantitis. The inclusion of diverse laser types, heterogeneous patient populations, and advanced microbiological techniques (including qPCR and 16S rRNA sequencing) enhances both the comprehensiveness and clinical relevance of the findings.

The literature search was exhaustive, encompassing electronic databases, grey literature, and manual searches up to February 2025. Risk of bias was systematically assessed, and the quality of evidence was evaluated using the GRADE framework. Furthermore, the use of a random-effects model in the meta-analysis accounted for significant between-study heterogeneity in interventions, populations, and outcomes.

Nonetheless, several limitations must be acknowledged. The most prominent is the substantial heterogeneity across included studies. Differences in laser type, wavelength, power settings, disease severity, treatment protocols, and comparator interventions introduced methodological variability that hindered data synthesis. Additionally, disparities in microbiological sampling and analysis methods—from culture-based techniques to molecular diagnostics—limited comparability and constrained meta-analytical aggregation to only a few bacterial species.

The high statistical heterogeneity observed (I^2^ > 75%) limits the interpretability of pooled estimates. Inconsistencies in follow-up durations, which ranged from 1 to 12 months, may have obscured insights into long-term treatment effects, such as microbial recolonization or relapse. Moreover, microbial outcomes were often not directly correlated with clinical or immunological endpoints, restricting the ability to evaluate overall therapeutic efficacy. Finally, small sample sizes in several trials reduced statistical power and further limited generalizability.

### 4.5. Future Directions

Future research should prioritize well-designed, multicenter RCTs employing standardized laser protocols, consistent microbiological and clinical endpoints, and long-term follow-up extending beyond 6 to 12 months. Efforts should be directed toward optimizing laser parameters, including energy settings, application duration, and delivery technique, specific to each modality.

Elucidating the underlying biological mechanisms of laser effects, including host modulation and inflammatory response, is also essential. Given the potential of PDT, particularly in medically compromised individuals or cases refractory to standard treatment, further investigation is warranted. PDT should be explored in combination with host-modulating agents or anti-inflammatory therapies to assess synergistic effects.

Advancements in microbiological diagnostics, such as metagenomic sequencing, microbial community profiling, and resistome analysis, offer promising tools for future studies. These techniques could deepen our understanding of microbial shifts and antibiotic resistance patterns, thus informing precision medicine approaches and antimicrobial stewardship in peri-implant therapy.

### 4.6. Conclusions

This review underscores the emerging, yet still inconclusive, role of laser-based adjunctive therapies in the non-surgical management of peri-implantitis from a microbiological standpoint. Current evidence does not support a consistent or statistically significant microbial advantage of laser-assisted approaches over conventional MD.

While certain modalities, PDT, show promise in reducing specific pathogenic bacteria in the short term, the lack of sustained effects, combined with methodological heterogeneity across studies, limits the strength of current recommendations. MD remains the foundational intervention for peri-implant disease management.

Within a personalized medicine framework, laser-based interventions—particularly PDT—may be considered in patient subgroups with systemic comorbidities, heightened inflammatory response, or contraindications to systemic antimicrobials. Individual risk profiling and microbial assessment should guide treatment selection. Future clinical trials should stratify outcomes by patient-specific characteristics to enable evidence-based personalized treatment protocols.

## Figures and Tables

**Table 1:** General characteristics of the included studies regarding microbial parameters. PIMD: peri-implant manual debridement; ICG-PDT: indocyanine green-mediated photodynamic therapy.

Authors, Year, Country	Study Design, Sample Size	Test Group	Control Group	Evaluated Bacteria	Bacteria Sampling Method and Analysis	Follow Ups	Results
1. Persson et al., 2011, Sweden [30]	Parallel, 42 patients/100 implants	21 patients/55 implants: Er: YAG laser (100 mJ/pulse, 10 Hz, 12.7 J/cm^2^, cone-shaped sapphire tip)	21 patients/45 implants: Air-abrasive device (Perio-flow, EMS), 15s per position covering full circumference	74 bacterial species	2 sterile paper points (size 55) placed at deepest PPD site for 20s. Checkerboard DNA-DNA hybridization.	1, 3, 6 months	No significant reduction in bacterial counts at 6 months in either group. Air-abrasive had short-term advantages (1 month) in reducing specific pathogens.
2. Arısan et al., 2015, Turkey [31]	Split-mouth, 10 patients/48 implants	24 implants: MD with plastic curette + diode laser (810 nm, 1 W, 3 J/cm^2^, pulsed mode, 1 min, spot diameter 1 mm, 400 μm fiber tip)	24 implants: MD with plastic curette only	20 species including *A. actinomycetemcomitans*, *P. gingivalis*, *T. denticola*	2 sterile paper points into peri-implant sulci for 20s, avoiding trauma-related bleeding. PCR and hybridization, semi-quantitative labeling scheme.	1 month	No significant changes in bacterial load at 1 month in either group. Diode laser showed no additional benefit compared to conventional scaling.
3. Labban et al., 2021, Saudi Arabia [34]	Parallel, 48 patients/64 implants	35 implants: ICG-PDT (repeated at 7, 17, 27 days) + PIMD (diode laser, 810 nm, 200 mW, 4 J, 30s at papilla, 10s in pocket)	29 implants: PIMD with ultrasonic device and carbon tip	*P. gingivalis*, *T. denticola*	Plaque collected from deepest peri-implant pocket, transferred to sterile microvials with PBS, vortexed. Detection of *P. gingivalis* and *T. denticola* on culture medium.	3, 6 months	ICG-PDT showed significant reduction in *P. gingivalis* and *T. denticola* at 3 and 6 months. PIMD effects diminished at 6 months.
4. Chen et al, 2021, Taiwan [33]	Parallel, 23 patients/25 implants	13 implants: Er: YAG laser repeated at 2 and 4 weeks (2940 nm, pulsed mode, 10 Hz, 100 mJ/pulse)	12 implants: MD with ultrasonic scaler	Anaerobic bacterial counts	Plaque collected using sterile paper points, transferred to transport medium. Anaerobic culture.	Post-tx, 3, 6 months	Test group showed no significant changes in anaerobic bacterial counts. Control group showed significant reduction at 3 and 6 months.
5. Tribble et al., 2022, USA [35]	Parallel, 38 patients	12 patients: non-surgical MD and aPDT (LLLT and a photosensitizer)	8 patients: non-surgical MD and non-activated aPDT	Total bacterial species including red/orange complex species	Plaque collected using sterile scaler, stored at −80°C. 16S rRNA gene amplification and sequencing for diversity and abundance.	3 months	Reduction in red complex pathogens in both groups, but not statistically significant. Minor shifts in bacterial species and abundance.
6. Roccuzzo et al., 2022, Switzerland [32]	Parallel, 25 patients/25 implants	12 patients: MD + diode laser repeated at 7 and 14 days (810 nm, 2.5 W, 50 Hz, 10 ms, applied 3x for 30s)	13 patients: MD + non-activated diode laser (repeated at 7 and 14 days)	*P. gingivalis*, *T. denticola*, *T. forsythia*, *F. nucleatum*, *C. rectus*	Sterile paper points placed at the bottom of the pocket at deepest PPD site. Multiplex real-time qPCR.	3, 6 months	Significant bacterial reduction in test and control groups at T1 (*P. gingivalis*, *T. forsythia*, *F. nucleatum*). At T2, only *F. nucleatum* showed significant reduction in control group.
7. Bassetti et al., 2013, Germany [37]	Parallel, 40 patients	20 patients: MD with titanium curettes, glycine-based powder air polishing, and PDT. Diode laser (660 nm, 100 mW, 10s) with phenothiazine chloride dye, repeated at 1 week and if residual BoP, treatment repeated at 3, 6, 9, and 12 months.	20 patients: MD with titanium curettes, glycine-based powder air polishing, and 1 mg minocycline microspheres locally into peri-implant pockets. If residual BoP, treatment repeated at 3, 6, 9, and 12 months.	*P. Gingivalis*, *T. forsythia*, *T. denticola*, *A. actinomycetemcomitans*, *P. intermedia*, *C. rectus*, *F. nucleatum*, *C. Gingivalis*, *P. micra*, *E. nodatum*, *E. corrodens*	Sterile paper points (ISO 055) for 15s from deepest PPD sites. DNA extraction with Chelex Method. Real-time PCR.	3, 6, 12 months	Both techniques were equally effective with no significant biofilm differences. At 3 months, PDT reduced *P. gingivalis*, *T. forsythia*, and *T. denticola*, while LDD reduced seven species. At 6 months, PDT reduced *P. gingivalis*, *T. forsythia*, and *F. nucleatum*, and LDD showed broader reductions. By 12 months, PDT showed minimal reduction, while LDD maintained significant decreases.
8. Almohareb et al., 2020, Saudi Arabia [36]	Parallel, 40 patients	20 patients: PDT with ultrasonic scaler and methylene blue mediated by diode laser (670 nm, 10s per site, 0.06 cm spot size) as adjunct to MD.	20 patients: MD with ultrasonic scaler and oral antibiotics (500 mg amoxicillin, 400 mg metronidazole, thrice daily for 7 days).	*P. gingivalis*, *T. denticola*, *T. forsythia*	Sterile paper points placed at the deepest PPD for 10s. PCR, identification, and calculation of bacterial content (Pg, Td, Tf) with the guidor Perio-Implant Diagnostic Test.	6, 12 months	Significant differences in *P. gingivalis*, *T. denticola*, and *T. forsythia* at 6 months compared to baseline for both groups. PDT showed a significant reduction in Pg and was as effective as antimicrobial therapy in reducing peri-implant symptoms.

**Table 2:** Quality of available evidence (GRADE assessment). [RCT: randomized controlled trial].

Outcome	Implants (Studies)	Study Design	Risk of Bias	Inconsistency	Indirectness	Imprecision	Publication Bias	Certainty
*F. nucleatum* counts	125 (2 RCTs)	RCT	Not serious	Serious	Serious	Serious	Unlikely	⬤◯◯◯ Very Low
*P. gingivalis* counts	189 (3 RCTs)	RCT	Not serious	Serious	Serious	Serious	Unlikely	⬤◯◯◯ Very Low
*T. denticola* counts	89 (2 RCTs)	RCT	Not serious	Serious	Serious	Serious	Unlikely	⬤◯◯◯ Very Low

## Data Availability

The original contributions presented in this study are included in the article and Supplementary Material. Further inquiries can be directed to the corresponding author.

## Supplementary Materials

The following supporting information can be downloaded at: https://www.mdpi.com/article/10.3390/dj14010049/s1, Figure S1. Additional meta-analytic data on *Fusobacterium nucleatum*. Figure S2. Additional meta-analytic data on *Porphyromonas gingivalis*. Figure S3. Additional meta-analytic data on *Treponema denticola*. Table S1. PRISMA 2020 checklist. Table S2. Eligibility criteria. Table S3. Detailed strategy for database search (up to 3 February 2025). Table S4. Additional information about the included studies. Table S5. Sensitivity analyses.

## Abbreviation

ANOVA	Analysis of Variance
RCT	Randomized Controlled Trials
Nd-YAG	Neodymium-doped yttrium aluminum garnet laser
Er-YAG	Erbium doped yttrium aluminum garnet laser
PDT	Photodynamic therapy
aPDT	Antimicrobial PDT
LLLT	Low-level laser therapy
MD	Mechanical debridement
LDD	Local drug delivery
BOP	Bleeding on probing
PI	Plaque index
PD	Probing depth
CAL	Clinical attachment level
REC	Gingival recession
KT	Width of keratinized tissue
MBL	Marginal bone loss
IL-1β, IL-6, IL-10	Interleukins
MMP-1, MMP-8	Matrix metalloproteinases
*F. nucleatum*	*Fusobacterium nucleatum*
*T. denticola*	*Tannerella forsythia*
*T. denticola*	*Treponema denticola*
*P. gingivalis*	*Porphyromonas gingivalis*
*E. nodatum*	*Eubacterium nodatum*
*P. intermedia*	*Prevotella intermedia*
PCR	Polymerase chain
Qpcr	Reaction quantitative real-time PCR
ICG	Indocyanine green
ITT analyses	Intention-to-treat analyses
SMDs	Standardized mean differences
GRADE	Grading of Recommendations Assessment, Development, and Evaluation
