# Supplementary material for: Microbiological Effects of Laser-Assisted Non-Surgical Treatment of Peri-Implantitis: A Systematic Review and Meta-Analysis of Randomized Controlled Trials
Source: Dent J (Basel). 2026 Jan 12;14(1):49. doi: 10.3390/dj14010049 (PMC12840075; doi:10.3390/dj14010049)
Supplement: Supplementary file 1 [file dentistry-14-00049-s001.zip › supplementary figures.pdf]

Supplementary Figures

Groups		Effect size and 95% confidence interval					Test of null (2-Tail)		Prediction Interval		Between-study		Other heterogeneity statistics			
Group	Number Studies	Point estimate	Standard error	Variance	Lower limit	Upper limit	Z-value	P-value	Lower limit	Upper limit	Tau	TauSq	Q-value	df (Q)	P-value	I-squared
**Fixed effect analysis**																
[0] Baseline	2	0.110	0.246	0.060	-0.372	0.592	0.448	0.654					1.283	1	0.257	22.073
[1] 3 months	2	-0.044	0.245	0.060	-0.525	0.436	-0.181	0.856					0.636	1	0.425	0.000
[2] 6 months	2	0.132	0.245	0.060	-0.348	0.613	0.539	0.590					0.497	1	0.481	0.000
Total within													2.417	3	0.491	
Total between													0.308	2	0.857	
Overall	6	0.066	0.142	0.020	-0.212	0.343	0.465	0.642					2.725	5	0.742	0.000
**Mixed effects analysis**																
[0] Baseline	2	0.127	0.283	0.080	-0.427	0.681	0.449	0.653			0.192	0.037				
[1] 3 months	2	-0.044	0.245	0.060	-0.525	0.436	-0.181	0.856			0.000	0.000				
[2] 6 months	2	0.132	0.245	0.060	-0.348	0.613	0.539	0.590			0.000	0.000				
Total between													0.323	2	0.851	
Overall	6	0.067	0.148	0.022	-0.223	0.356	0.451	0.652			0.000	0.000				

Figure S1: Additional meta-analytic data on *Fusobacterium nucleatum.*

Groups		Effect size and 95% confidence interval					Test of null (2-Tail)		Prediction Interval		Between-study		Other heterogeneity statistics			
Group	Number Studies	Point estimate	Standard error	Variance	Lower limit	Upper limit	Z-value	P-value	Lower limit	Upper limit	Tau	TauSq	Q-value	df (Q)	P-value	I-squared
**Fixed effect analysis**																
[0] Baseline	3	0.005	0.189	0.036	-0.364	0.375	0.028	0.977					4.866	2	0.088	58.895
[1] 3 months	3	-0.837	0.212	0.045	-1.253	-0.421	-3.941	0.000					36.951	2	0.000	94.587
[2] 6 months	3	-0.512	0.195	0.038	-0.894	-0.130	-2.627	0.009					12.521	2	0.002	84.026
Total within													54.337	6	0.000	
Total between													9.162	2	0.010	
Overall	9	-0.416	0.114	0.013	-0.640	-0.192	-3.642	0.000					63.499	8	0.000	87.401
**Mixed effects analysis**																
[0] Baseline	3	0.058	0.300	0.090	-0.529	0.646	0.195	0.846	-6.270	6.386	0.398	0.158				
[1] 3 months	3	-1.034	0.933	0.871	-2.863	0.796	-1.107	0.268	-24.252	22.185	1.571	2.468				
[2] 6 months	3	-0.476	0.494	0.244	-1.444	0.492	-0.964	0.335	-12.239	11.287	0.783	0.613				
Total between													1.815	2	0.404	
Overall	9	-0.152	0.247	0.061	-0.636	0.332	-0.615	0.538	-2.375	2.071	0.907	0.822				

Figure S2: Additional meta-analytic data on *Porphyromonas gingivalis*.

Groups		Effect size and 95% confidence interval					Test of null (2-Tail)		Prediction Interval		Between-study		Other heterogeneity statistics			
Group	Number Studies	Point estimate	Standard error	Variance	Lower limit	Upper limit	Z-value	P-value	Lower limit	Upper limit	Tau	TauSq	Q-value	df (Q)	P-value	I-squared
**Fixed effect analysis**																
[0] Baseline	2	0.103	0.235	0.055	-0.358	0.564	0.438	0.662					1.265	1	0.261	20.971
[1] 3 months	2	-1.645	0.348	0.121	-2.328	-0.962	-4.721	0.000					57.751	1	0.000	98.268
[2] 6 months	2	-1.384	0.298	0.089	-1.968	-0.800	-4.644	0.000					34.237	1	0.000	97.079
Total within													93.253	3	0.000	
Total between													24.241	2	0.000	
**Random effects analysis**																
[0] Baseline	2	0.085	0.270	0.073	-0.445	0.614	0.313	0.754			0.181	0.033				
[1] 3 months	2	-3.196	3.082	9.501	-9.237	2.846	-1.037	0.300			4.321	18.675				
[2] 6 months	2	-1.559	1.753	3.072	-4.994	1.876	-0.889	0.374			2.442	5.964				
Total between													1.957	2	0.376	

Figure S3: Additional meta-analytic data on *Treponema denticola.*
