# Supplementary material for: Microbiological Effects of Laser-Assisted Non-Surgical Treatment of Peri-Implantitis: A Systematic Review and Meta-Analysis of Randomized Controlled Trials
Source: Dent J (Basel). 2026 Jan 12;14(1):49. doi: 10.3390/dj14010049 (PMC12840075; doi:10.3390/dj14010049)
Supplement: Supplementary file 1 [file dentistry-14-00049-s001.zip › supplementary tables.pdf]

**Supplementary Tables**

**Table S1:PRISMA 2020 checklist:**

| Section and Topic | Item # | Checklist item | Location where item is reported |
|---|---|---|---|
| | | **TITLE** | |
| Title | 1 | Identify the report as a systematic review. | 1 |
| | | **ABSTRACT** | |
| Abstract | 2 | See the PRISMA 2020 for Abstracts checklist. | 2 |
| | | **INTRODUCTION** | |
| Rationale | 3 | Describe the rationale for the review in the context of existing knowledge. | 3 |
| Objectives | 4 | Provide an explicit statement of the objective(s) or question(s) the review addresses. | 4 |
| | | **METHODS** | |
| Eligibility criteria | 5 | Specify the inclusion and exclusion criteria for the review and how studies were grouped for the syntheses. | 4, Table 1 |
| Information sources | 6 | Specify all databases, registers, websites, organisations, reference lists and other sources searched or consulted to identify studies. Specify the date when each source was last searched or consulted. | 4, Suppl. Table A1 |
| Search strategy | 7 | Present the full search strategies for all databases, registers and websites, including any filters and limits used. | 7, Suppl. Table A1 |
| Selection process | 8 | Specify the methods used to decide whether a study met the inclusion criteria of the review, including how many reviewers screened each record and each report retrieved, whether they worked independently, and if applicable, details of automation tools used in the process. | 4-5 |
| Data collection process | 9 | Specify the methods used to collect data from reports, including how many reviewers collected data from each report, whether they worked independently, any processes for obtaining or confirming data from study investigators, and if applicable, details of automation tools used in the process. | 4-5 |
| Data items | 10a | List and define all outcomes for which data were sought. Specify whether all results that were compatible with each outcome domain in each study were sought (e.g. for all measures, time points, analyses), and if not, the methods used to decide which results to collect. | 4-5 |
| | 10b | List and define all other variables for which data were sought (e.g. participant and intervention characteristics, funding sources). Describe any assumptions made about any missing or unclear information. | 4-5 |
| Study risk of bias assessment | 11 | Specify the methods used to assess risk of bias in the included studies, including details of the tool(s) used, how many reviewers assessed each study and whether they worked independently, and if applicable, details of automation tools used in the process. | 5 |
| Effect measures | 12 | Specify for each outcome the effect measure(s) (e.g. risk ratio, mean difference) used in the synthesis or presentation of results. | 5 |
| Synthesis methods | 13a | Describe the processes used to decide which studies were eligible for each synthesis (e.g. tabulating the study intervention characteristics and comparing against the planned groups for each synthesis (item #5)). | 5 |
| | 13b | Describe any methods required to prepare the data for presentation or synthesis, such as handling of missing summary statistics, or data conversions. | 5 |
| | 13c | Describe any methods used to tabulate or visually display results of individual studies and syntheses. | 5 |
| | 13d | Describe any methods used to synthesize results and provide a rationale for the choice(s). If meta-analysis was performed, describe the model(s), method(s) to identify the presence and extent of statistical heterogeneity, and software package(s) used. | 5 |
| | 13e | Describe any methods used to explore possible causes of heterogeneity among study results (e.g. subgroup analysis, meta- | 5 |

| Section and Topic | Item # | Checklist item | Location where item is reported |
|---|---|---|---|
| | | regression). | |
| | 13f | Describe any sensitivity analyses conducted to assess robustness of the synthesized results. | 5 |
| Reporting bias assessment | 14 | Describe any methods used to assess risk of bias due to missing results in a synthesis (arising from reporting biases). | 5 |
| Certainty assessment | 15 | Describe any methods used to assess certainty (or confidence) in the body of evidence for an outcome. | 5 |
| **RESULTS** | | | |
| Study selection | 16a | Describe the results of the search and selection process, from the number of records identified in the search to the number of studies included in the review, ideally using a flow diagram. | 6, Fig 2 |
| | 16b | Cite studies that might appear to meet the inclusion criteria, but which were excluded, and explain why they were excluded. | 6, Suppl. Table A2 |
| Study characteristics | 17 | Cite each included study and present its characteristics. | 6, Table 2 |
| Risk of bias in studies | 18 | Present assessments of risk of bias for each included study. | 6-8, Fig 3 |
| Results of individual studies | 19 | For all outcomes, present, for each study: (a) summary statistics for each group (where appropriate) and (b) an effect estimate and its precision (e.g. confidence/credible interval), ideally using structured tables or plots. | 8-9 |
| Results of syntheses | 20a | For each synthesis, briefly summarise the characteristics and risk of bias among contributing studies. | 8-9 |
| | 20b | Present results of all statistical syntheses conducted. If meta-analysis was done, present for each the summary estimate and its precision (e.g. confidence/credible interval) and measures of statistical heterogeneity. If comparing groups, describe the direction of the effect. | 8-9 |
| | 20c | Present results of all investigations of possible causes of heterogeneity among study results. | 8-9 |
| | 20d | Present results of all sensitivity analyses conducted to assess the robustness of the synthesized results. | 8, Suppl. Table 4 |
| Reporting biases | 21 | Present assessments of risk of bias due to missing results (arising from reporting biases) for each synthesis assessed. | 9 |
| Certainty of evidence | 22 | Present assessments of certainty (or confidence) in the body of evidence for each outcome assessed. | 9, Table 3 |
| **DISCUSSION** | | | |
| Discussion | 23a | Provide a general interpretation of the results in the context of other evidence. | 10 |
| | 23b | Discuss any limitations of the evidence included in the review. | 11 |
| | 23c | Discuss any limitations of the review processes used. | 11 |
| | 23d | Discuss implications of the results for practice, policy, and future research. | 12 |
| **OTHER INFORMATION** | | | |
| Registration and protocol | 24a | Provide registration information for the review, including register name and registration number, or state that the review was not registered. | 4 |
| | 24b | Indicate where the review protocol can be accessed, or state that a protocol was not prepared. | 4 |
| | 24c | Describe and explain any amendments to information provided at registration or in the protocol. | N/A |
| Support | 25 | Describe sources of financial or non-financial support for the review, and the role of the funders or sponsors in the review. | 12 |

| Section and Topic | Item # | Checklist item | Location where item is reported |
|---|---|---|---|
| Competing interests | 26 | Declare any competing interests of review authors. | 12 |
| Availability of data, code and other materials | 27 | Report which of the following are publicly available and where they can be found: template data collection forms; data extracted from included studies; data used for all analyses; analytic code; any other materials used in the review. | 12 |

**Table S2.** Eligibility criteria.

| Domain | Inclusion criteria | Exclusion criteria |
|---|---|---|
| **Participants** | ▪ Adult patients with at least one implant diagnosed with peri-implantitis (according to any approved classification to date). | |
| **Interventions** | ▪ Non-surgical treatment of peri-implantitis using various types of lasers (diode, erbium, Nd:YAG, PDT, LLLT) as monotherapy or adjunctive to mechanical debridement. | |
| **Comparisons** | ▪ Conventional non-surgical treatment of peri-implantitis (mechanical debridement) using plastic/titanium curettes, ultrasonic scalers, air abrasive devices (air polishing), or combinations, with or without local/systemic antimicrobials. | |
| **Outcomes** | ▪ Microbiological changes, including qualitative or quantitative shifts in peri-implant pathogens. | |
| **Study design** | ▪ Randomized clinical trials with at least 1 month of observation period after the intervention and a sample size of at least 10 patients. | ▪ Animal, in vitro, ex-vivo, or in silico studies.<br>▪ Non-comparative studies (case reports and case series).<br>▪ Cohort and case-control studies.<br>▪ Non-randomized studies.<br>▪ Systematic reviews and meta-analyses. |

**Table S3.** Detailed strategy for database search (up to February 3rd, 2025).

| Database [2025 02 03] | Search strategy | Hits |
|---|---|---|
| **General Sources** | | |
| **PubMed** | (“peri implantitis” OR peri-implantitis OR periimplantitis) AND (laser OR “Er:YAG” OR erbium OR “Nd:YAG” OR “Er,Cr:YSGG” OR neodymium-doped OR CO2 OR diode OR PDT OR photodynamic) AND (bacteria OR biofilm OR bacterial OR microbia OR microorganisms OR microbiologic OR microbiota OR microbiological OR “a.a” OR porphyromonas OR gingivalis OR anaerobic OR actinobacillus OR actinomycetemcomitans OR prevotella OR intermedia OR tannerella OR forsythia OR treponema OR denticola OR fusobacterium OR nucleatum OR streptococcus) | **314** |
| **Cochrane Central Register of Controlled Trials** | (“peri implantitis” OR peri-implantitis OR periimplantitis) AND (laser OR erbium OR neodymium OR CO2 OR diode OR PDT OR photodynamic) AND (bacteria OR biofilm OR bacterial OR microbia OR microorganisms OR microbiologic OR microbiota OR microbiological OR “a.a” OR porphyromonas OR gingivalis OR anaerobic OR actinobacillus OR actinomycetemcomitans OR prevotella OR intermedia OR tannerella OR forsythia OR treponema OR denticola OR fusobacterium OR nucleatum OR streptococcus) in Title Abstract Keyword - (Word variations have been searched) | **80** |
| **Cochrane Database of Systematic Reviews** | (“peri implantitis” OR peri-implantitis OR periimplantitis) AND (laser OR erbium OR neodymium OR CO2 OR diode OR PDT OR photodynamic) AND (bacteria OR biofilm OR bacterial OR microbia OR microorganisms OR microbiologic OR microbiota OR microbiological OR “a.a” OR porphyromonas OR gingivalis OR anaerobic OR actinobacillus OR actinomycetemcomitans OR prevotella OR intermedia OR tannerella OR forsythia OR treponema OR denticola OR fusobacterium OR nucleatum OR streptococcus) in Title Abstract Keyword - (Word variations have been searched) | **1** |
| **Scopus** | TITLE-ABS ((“peri implantitis” OR peri-implantitis OR periimplantitis) AND (laser OR “Er:YAG” OR erbium OR “Nd:YAG” OR “Er,Cr:YSGG” OR neodymium-doped OR CO2 OR diode OR PDT OR photodynamic) AND (bacteria OR biofilm OR bacterial OR microbia OR microorganisms OR microbiologic OR microbiota OR microbiological OR “a.a” OR porphyromonas OR gingivalis OR anaerobic OR actinobacillus OR actinomycetemcomitans OR prevotella OR intermedia OR tannerella OR forsythia OR treponema OR denticola OR fusobacterium OR nucleatum OR streptococcus)) | **255** |
| **Web of Science™ Core Collection**<br><br>http://apps.webofknowledge.com/ | (“peri implantitis” OR peri-implantitis OR periimplantitis) AND (laser OR “Er:YAG” OR erbium OR “Nd:YAG” OR “Er,Cr:YSGG” OR neodymium-doped OR CO2 OR diode OR PDT OR photodynamic) AND (bacteria OR biofilm OR bacterial OR microbia OR microorganisms OR microbiologic OR microbiota OR microbiological OR “a.a” OR porphyromonas OR gingivalis OR anaerobic OR actinobacillus OR actinomycetemcomitans OR prevotella OR intermedia OR tannerella OR forsythia OR treponema OR denticola OR fusobacterium OR nucleatum OR streptococcus) (Title) OR (“peri implantitis” OR peri-implantitis OR periimplantitis) AND (laser OR “Er:YAG” OR erbium OR “Nd:YAG” OR “Er,Cr:YSGG” OR neodymium-doped OR CO2 OR diode OR PDT OR photodynamic) AND (bacteria OR biofilm OR bacterial OR microbia OR microorganisms OR microbiologic OR microbiota OR microbiological OR “a.a” OR porphyromonas OR gingivalis OR anaerobic OR actinobacillus OR actinomycetemcomitans OR prevotella OR intermedia OR tannerella OR forsythia OR treponema OR denticola OR fusobacterium OR nucleatum OR streptococcus) (Abstract) and Preprint Citation Index (Exclude – Database) | **274** |

| | | |
|---|---|---|
| | All databases. Timespan: All years. Search language=Auto | |
| **ProQuest Dissertations and Theses Global** | title((“peri implantitis” OR peri-implantitis OR periimplantitis) AND (laser OR “Er:YAG” OR erbium OR “Nd:YAG” OR “Er,Cr:YSGG” OR neodymium-doped OR CO2 OR diode OR PDT OR photodynamic) AND (bacteria OR biofilm OR bacterial OR microbia OR microorganisms OR microbiologic OR microbiota OR microbiological OR “a.a” OR porphyromonas OR gingivalis OR anaerobic OR actinobacillus OR actinomycetemcomitans OR prevotella OR intermedia OR tannerella OR forsythia OR treponema OR denticola OR fusobacterium OR nucleatum OR streptococcus)) OR abstract((“peri implantitis” OR peri-implantitis OR periimplantitis) AND (laser OR “Er:YAG” OR erbium OR “Nd:YAG” OR “Er,Cr:YSGG” OR neodymium-doped OR CO2 OR diode OR PDT OR photodynamic) AND (bacteria OR biofilm OR bacterial OR microbia OR microorganisms OR microbiologic OR microbiota OR microbiological OR “a.a” OR porphyromonas OR gingivalis OR anaerobic OR actinobacillus OR actinomycetemcomitans OR prevotella OR intermedia OR tannerella OR forsythia OR treponema OR denticola OR fusobacterium OR nucleatum OR streptococcus))<br><br>Filters activated: Full text | **25** |
| **Total** | | **949** |

**Table S4.** Additional information about the included studies.

| Authors, Year | Inclusion criteria | Other parameters evaluated besides microbial | Contribution/Funding | Universities | Open access |
|---|---|---|---|---|---|
| 1. Persson et al., 2011 | ≥1 implant with radiographic bone loss ≥2 mm, PPD ≥5 mm with BOP or pus on probing | PPD, BOP, SUP, radiographic changes, treatment outcome | Supported by University of Bern, EMS (Electro Medical Systems, Switzerland), KAVO (Germany), and Philips (USA). | Kristianstad University, Sweden | No |
| 2. Arısan et al., 2015 | ≥2 functioning bilateral rough-surfaced implants, PPD 4–6 mm, <3 mm MBL, with BOP, plaque, pain, or suppuration | PPD, PI, BoP, MBL radiographically | Funded by Istanbul University Research Fund (No: 22148). | Faculty of Dentistry, Istanbul University, Turkey | Yes |
| 3. Labban et al., 2021 | >45 years, type 2 DM (HbA1c ≥6.5%), PPD ≥6 mm with BOP or suppuration, alveolar bone loss ≥3 mm | PPD, BoP, Sup, radiographic PCBL, IL-1β, IL-6 | No funding details provided. | College of Dentistry, King Saud University, Saudi Arabia | Yes |
| 4. Chen et al, 2021 | ≥1 implant with peri-implantitis, in function ≥6 months, vertical bone loss, PPD <7 mm with BOP | PPD, BOP, radiographic MBL | Supported by Southern Taiwan Science Park (Grant No: BX-04–11-21–106). | Kaohsiung Medical University Hospital, Taiwan | No |
| 5. Tribble et al., 2022 | ≥1 implant diagnosed with peri-implantitis, ≥18 years, systemically healthy or controlled systemic conditions | PPD, BOP, PI, KT | No funding details provided. | University of Texas Health Science Center, USA | Yes |
| 6. Roccuzzo et al., 2022 | ≥18 years, systemic health or controlled conditions, SLA implants, PPD >5 mm, BOP or suppuration, bone loss ≥2 mm, accessible for plaque control, ≥2 mm KM | PPD, BOP, Sup, PI, KM, radiographic bone changes, treatment success, IL-1β, IL-10, MMP-8 | Supported by a small grant from the International Team for Implantology (ITI) (No: 1374-2019). | University of Bern, Switzerland | No |
| 7. Bassetti et al., 2013, Germany | ≥18 years, no relevant medical conditions, healthy or treated periodontal conditions in supportive care, peri-implantitis with PPD 4–6 mm, BoP at 1 site, bone loss 0.5-2 mm, implant in function ≥1 year, SLA titanium implants, FMPS ≤25, FMBS ≤25 | BoP, PPD, CAL, REC, mPlI, IL-1b, IL-8, IL-10, MMP-1, MMP-8 | Supported by Bredent Medical GmbH & Co. KG, Geschäftsbereich HELBO, Walldorf, Germany. | University of Bern, Switzerland and private practice | No |
| 8. Almohareb et al., 2020, | ≥25 years, severe peri-implantitis with abscess, PD ≥6 mm on at least one implant, bone loss ≥3 | PS, PD, BoP, CAL, pain | Supported by the New Faculty Grants Program (RAÀD), The Deanship of | College of Dentistry, King Saud | No |

| | | | | | |
|---|---|---|---|---|---|
| Saudi Arabia | mm, CAL ≤3 mm, systemically healthy or controlled conditions | | Scientific Research, King Saud University. | University, Saudi Arabia | |

DM: Diabetes Mellitus; HbA1c: Hemoglobin A1c;

**Diabetic vs. Non-diabetic**

| | | Q-value | df (Q) | P-value | |
|---|---|---|---|---|---|
| *P.gingivalis* | [1] 3 months | 36,214 | 1 | 0.000 | More pronounced effects in the diabetic patients |
| *P.gingivalis* | [2] 6 months | 12,518 | 1 | 0.000 | More pronounced effects in the diabetic patients |

**Air-abrasion vs. Mechanical debridement**

| | | Q-value | df (Q) | P-value |
|---|---|---|---|---|
| *P.gingivalis* | [1] 3 months | 0.511 | 1 | 0.475 |
| *P.gingivalis* | [2] 6 months | 0.832 | 1 | 0.362 |

Type of laser treatment

| | | Q-value | df (Q) | P-value | |
|---|---|---|---|---|---|
| *P.gingivalis* | [1] 3 months | 36,951 | 2 | 0.000 | More pronounced effects in the PDT therapy study [potentially confounded by the diabetic medical history of patients] |
| *P.gingivalis* | [2] 6 months | 12,521 | 2 | 0.002 | More pronounced effects in the PDT therapy study [potentially confounded by the diabetic medical history of patients] |

Table S5: Sensitivity analyses.
